# 

**Published:** 1896-06-20

**Authors:** 


**Notices of Births, Deaths, and Marriages** are inserted in this column at a charge of 2s. 6d. for an announcement not exceeding four lines.

**Notice to Advertisers and Subscribers.**
All Advertisements, Orders for Copies of The Hospital and Business Communications should be addressed to **THE MANAGER, (Not to The Editor),** The Hospital, 428, Strand, London, W.C.

The Cost of Subscription, post free, is as follows:—Per week, 2½d.; per quarter, 2s. 8d.; per half-year, 5s. 3d.; per annum, 10s. 6d. Foreign:—Per Annum, 15s. 2d.; payable, in all cases, in advance.

Now Ready.
**"Burdett's Hospitals and Charities, 1896."**
Seventh year of publication. Over 1,000 pp. Scarlet cloth, gilt lettered, Price 5s. A most complete guide to British, Colonial, Indian, and American Hospitals, Dispensaries, Nursing and Convalescent Institutions, and Asylums. A few complete sets of the preceding six issues of the "Annual" may still be obtained on application to the Publishers, The Scientific Press (Limited), 428, Strand, London, W.C.

### NOTICE TO CORRESPONDENTS.

All MS., Letters, Books for Review, and other matters intended for the Editor should be addressed The Editor, The Lodge, Porchester Square, London, W.

The Editor cannot undertake to return rejected MS., even when accompanied by stamped directed envelope.

**NOTICE.—Vol. XIX. of "The Hospital" (October, 1895, to March, 1896), in handsome cloth binding, is now ready and on sale at the Office of this Journal. Price 7s. 6d. Cases for binding the Numbers contained in this Volume, 1s. 6d. Index to Vol. XIX., 2½d., post free.**
**The Monthly Part for June is now ready, price 6d., by post, 8d.**

### BOOKS RECEIVED.

DAVID NUTT.

"The Frog: An Introduction to Anatomy, Histology, and Embryology." By the late A. Milnes Marshall, M.D., edited by G. Herbert Fowler.

W. AND R. CHAMBERS (LIMITED).

"Elementary Human Physiology." By J. G. McKendrick, M.D., F.R.S.

SAXON AND CO.

"Everybody's Bible Dictionary." Edited by Walter Baches.

G. BELL AND SONS.

"Biological Experimentations." By Sir Benjamin Ward Richardson.

BALLIERE, TINDALL, AND COX.

"History of Surgical Anæsthesia." By H. Bellamy Gardner, M.R.C.S., L.R.C.P.

"Diagnosis and Treatment of Diseases of the Rectum." By W. Allingham, F.R.C.S., and Herbert Allingham, F.R.C.S.

J. AND A. CHURCHILL.

"Sterility." By Robert Bell, M.D.

"Southall's Materia Medica." Fifth and enlarged edition. By John Barclay.

Scientific Press (Limited).

"Elementary Anatomy and Surgery for Nurses." By W. McAdam Eccles, M.S.Lond., F.R.C.S.Eng.

Kegan Paul, Trench, Trubner, and Co.

"Infantile Mortality during Childbirth, and its Prevention." By A. Brothers, B.S., M.D.

**Periodicals and Pamphlets.**—Monthly Homœopathic Review, Practitioner, Inter-colonial Medical Journal of Australasia, Die Medizinische Litteratur, Leeds Hospital Magazine, Medical Mission Quarterly, Australasian Medical Gazette. Cornhill Magazine, International Medical Magazine, Cassell's Family Magazine, Yale Medical Journal, Charity Organisation Review, Medical Times, Charlotte Medical Journal, Nursing Record, Tri-State Medical Journal, London, New York Medical Journal, Medical Press, Pediatrics, Literary Digest, Medical Week, Therapist, Planter, Industries and Iron, Electrical Review, Sun, Scalpel, Invention, Clinical Journal, Sunday at Home, Leisure Hour, Girl's Own Paper, Boy's Own Paper, Columbus Medical Journal, Summer Tours in Scotland by David MacBrayne's Royal Mail Steamers.

### THE STUDENT OF MEDICINE.

### APPOINTMENTS.

A. J. Adkins, M.D., M.R.C.S., L.R.C.P., appointed Assistant Medical Officer and Dispenser at the Lambeth Union Workhouse. J. G. Bain, M.B., C.M.Glasg., appointed Medical Officer for the Second District of the Wellington (Som) Union. H. C. Bartlett, L.R.C.P.Lond., M.R.C.S., appointed Medical Officer for the Sixth District of the Saffron Walden Union. G. de Veulle Belson, M.A.Oxon., M.R.C.S., L.R.C.P., appointed Medical Officer of Health to the Bampton Urban District Council. T. A. Bowes, M.R.C.S.Eng., L.S.A., appointed an Honorary Medical Officer to the Herne Bay Cottage Hospital. R. O. Bowman, M.D.Lond., M.B., M.R.C.S., appointed Medical Officer for the Ulverston District of the Ulverston Union. R. J. Carter, M.D.Lond., D.P.H., appointed Assistant Physician to the Western Skin Hospital, London, W. S. W. Thomson, M.B., Ch.B.Vict., appointed House Surgeon to the Stockport Infirmary.

### VACANCIES.

The following vacancies are announced this week, the amount of salary in each case being given after the name of the institution:—

### Resident Medical Officers.

Central London Ophthalmic Hospital, 238A, Gray's Inn Road, W.C.—House Surgeon. Applications to the Secretary before July 7th.

General Hospital, Barbadoes.—Junior Resident Surgeon. Appointment for three years. Salary £200 per annum, payable monthly, with unfurnished quarters. Passage paid. Applications to the Secretary, Barbadoes General Hospital, by July 8th.

Lewes Dispensary and Infirmary, and Victoria Hospital, Lewes.—Resident Medical Officer; doubly qualified. Salary, £110 per annum, with furnished apartments, coal, gas, and attendance. Applications to R. Blaker, Honorary Secretary, by June 20th.

Chester General Infirmary.—Visiting Surgeon; must be doubly qualified. Appointment for not more than two years. Salary to commence at £90 per annum, with board and residence. Applications to the Chairman of the Board of Management, Secretary's Office, 29, Eastgate Row, North Chester, by June 27th.

Royal Free Hospital, Gray's Inn Road, W.C.—House Surgeon; doubly qualified. Appointment for six months, but the holder is eligible for re-election for a further period of six months. No salary, but board, residence, and washing provided. Applications to C. W. Thies, Secretary, by June 27th.

Seamen's Hospital Society (Dreadnought), Greenwich, S.E.—House Surgeon for the Branch Hospital, Royal Albert and Victoria Docks, E. Salary £75 per annum, with board and residence; must be doubly qualified. Applications to P. Michelli, Secretary, by June 30th.

Stockport Infirmary.—Assistant Surgeon and Visiting Surgeon; doubly qualified. Salary £70 per annum, with board, washing, and residence. Applications to the Secretary by June 23rd.

West London Hospital, Hammersmith Road, W.—Physician for Diseases of Women. Assistant Physician for Diseases of Women; must be for F. or M.R.C.P. Lond. House Physician and House Surgeon. Appointments for six months, board and lodging provided. Applications to R. J. Gilbert, Secretary-Superintendent, by June 24th.

Wolverhampton and Staffordshire General Hospital, Wolverhampton.—Resident Assistant. Appointment for six months. Board, lodging, and washing provided. Applications, inscribed "Applications for Resident Assistant," to be addressed to the Chairman of the Medical Committee by June 29th.

### Non-Resident Medical Officers.

East Dulwich Provident Dispensary.—Vacancy on the Medical Staff. Applications to the Secretary by June 20th.

Evelina Hospital for Sick Children, Southwark, S.E.—Four qualified Clinical Assistants and eight unqualified Clinical Clerks in the Out-patient Department. Applications to the Honorary Secretary of the Medical Committee by June 21st.

Grosvenor Hospital for Women and Children, Vincent Square, Westminster.—Dispenser. Application to the Secretary by June 20th.

Longmore Hospital for Incurables, Edinburgh.—Assistant Medical Officer. Resident in the immediate neighbourhood indispensable. Applications to the Secretary, 6, North Street, David Street, Edinburgh, by June 25th.

St. George's and St. James's Dispensary, 60, King Street, Regent Street, W.—Surgeon; must be M. or F.R.C.S.Eng. Applications to St. Leger Bunnett, Secretary, by June 20th.

**Medical Appointment.**

SEAMEN'S HOSPITAL SOCIETY (Dreadnought).

WANTED, HOUSE SURGEON for Branch Hospital, Royal Victoria and Albert Docks, E.

Salary £75 per annum, with Board and Residence.

